# Enamel Surface and Elemental Changes Following In Vitro Bleaching: A SEM-EDS Approach

**DOI:** 10.3390/dj13090431

**Published:** 2025-09-17

**Authors:** Berivan Laura Rebeca Buzatu, Ramona Dumitrescu, Magda Mihaela Luca, Roxana Buzatu, Atena Galuscan, Vanessa Bolchis, Gabriela Vlase, Titus Vlase, Daniela Elisabeta Jumanca

**Affiliations:** 1 Faculty of Dental Medicine, Doctoral School, “Victor Babes” University of Medicine and Pharmacy Timisoara, 300041 Timisoara, Romania; berivan.buzatu@umft.ro; 2 Translational and Experimental Clinical Research Centre in Oral Health, Faculty of Dental Medicine, “Victor Babes” University of Medicine and Pharmacy Timisoara, 300041 Timisoara, Romania; dumitrescu.ramona@umft.ro (R.D.); galuscan.atena@umft.ro (A.G.); vanessa.bolchis@umft.ro (V.B.); jumanca.daniela@umft.ro (D.E.J.); 3 Department of Preventive, Community Dentistry and Oral Health, Faculty of Dental Medicine, “Victor Babes” University of Medicine and Pharmacy Timisoara, 300041 Timisoara, Romania; 4 Pediatric Dentistry Research Center (Pedo-Research), Department of Pediatric Dentistry, Faculty of Dental Medicine, “Victor Babes” University of Medicine and Pharmacy Timisoara, 300041 Timisoara, Romania; 5 Department of Dental Aesthetics, Faculty of Dental Medicine, “Victor Babes” University of Medicine and Pharmacy Timisoara, 300041 Timisoara, Romania; 6 Research Centre for Thermal Analysis in Environmental Problems (ICAM), West University of Timisoara, 300115 Timisoara, Romania; gabriela-vlase@e-uvt.ro (G.V.); titus.vlase@e-uvt.ro (T.V.)

**Keywords:** tooth bleaching, tooth enamel/chemistry, hydrogen peroxide/pharmacology, microscopy, electron, scanning, X-Ray microanalysis

## Abstract

**Background and Objectives:** Chairside bleaching can alter enamel morphology and mineral content. This in vitro study compared surface changes and elemental shifts after three in-office protocols using scanning electron microscopy (SEM) and energy-dispersive X-ray spectroscopy (EDS). **Materials and Methods:** Forty-two human premolars/molars were sectioned; matched halves served as control or received Opalescence Quick 45% carbamide peroxide (CP), Opalescence Boost 40% hydrogen peroxide (HP), or BlancOne Ultra+ 35% HP with light activation. Gels were applied per manufacturers’ instructions. SEM assessed topography (×500–×1100); EDS quantified atomic percent of O, Ca, P, C and trace elements. One-way ANOVA compared Ca and P between bleached groups (α = 0.05). **Results:** Controls showed compact surfaces with preserved Ca and P. After Quick, SEM revealed roughness, fissures and microcracks; Ca fell from 11.5 to 12.5 to 9.53–11.73 at% and P from 7.5 to 8.9 to 7.41–8.59 at%. Boost produced mild superficial restructuring and granular deposits with variable Ca 13.80–27.94 at% and P 7.32–14.65 at%. BlancOne Ultra+ caused diffuse erosion and loss of prismatic clarity with marked demineralization (Ca 1.42–7.85 at%, P 1.22–6.71 at%); C rose locally to 46.61 at%. Across bleached groups, Ca and P differed significantly (both *p* < 0.001). Oxygen remained dominant (~39–50 at%) in all spectra; occasional Al/Si/Cl/K likely reflected residues or preparation artifacts. **Conclusions:** All protocols produced surface and compositional alterations, with a severity gradient: BlancOne Ultra+ > Boost > Quick. High-concentration, light-activated HP yielded the largest Ca/P losses. Clinically, neutral-pH, non-activated or chemically activated regimens and immediate post-bleach remineralization should ideally be used when feasible, particularly before adhesive procedures.

## 1. Introduction

Tooth bleaching has become one of the most requested aesthetic procedures, driven by social visibility and the expansion of both professional and at-home products. Chemically, whitening relies on the diffusion of hydrogen peroxide (HP) or carbamide peroxide (CP) through enamel and into dentin, where reactive oxygen species oxidize chromogens and lighten tooth color; efficacy scales mainly with concentration and exposure time, although lower concentrations can approach higher-dose outcomes if applied longer [1,2,3]. While clinical effectiveness is well documented, reviews emphasize that safety must be balanced against transient side effects (hypersensitivity, gingival irritation) and possible enamel changes, especially with high-concentration, in-office protocols [2,4,5,6,7,8].

Several material and procedural variables modulate enamel responses. Beyond dose and time, peroxide release kinetics and gel formulation govern how much active HP reaches the surface and penetrates tissues [5,6,9,10,11]. Surface pH is equally important: acidic gels can enhance oxidative action but also increase mineral solubility, whereas pH conditioners can maintain efficacy while mitigating demineralization [12,13,14]. Light/heat activation may accelerate radical generation, yet its added clinical benefit remains inconsistent across studies and device types, leading many authors to recommend cautious, evidence-based use rather than routine photoactivation [1,2,3,4]. These uncertainties justify controlled, head-to-head comparisons of prevalent chairside systems.

Morphological outcomes reported by SEM range from subtle prism-sheath accentuation and porosity to surface roughening and shallow erosive features; the magnitude varies by protocol and storage medium post-treatment (e.g., artificial saliva can promote precipitation/redeposition) [6,7,8]. Concomitantly, mechanical surrogates (microhardness/roughness) often shift after bleaching, again with protocol-dependent direction and size of effect [11,12]. Notably, meta-analytic data suggest that typical at-home 10% CP regimens may not meaningfully reduce enamel microhardness, underscoring how concentration and application regimen shape risk [12].

Elemental analyses help link morphology to composition. EDS studies show that high-concentration HP, especially in repeated or condensed sessions, can locally reduce Ca and P, lowering the Ca/P ratio; in some models, remineralizing components (e.g., added calcium, phosphate, or post-treatment agents) partially restore the profile without compromising color change [9,10,11,13,14]. These findings position EDS (paired with SEM) as a pragmatic endpoint for quantifying early surface chemical shifts that precede or accompany textural change. Given the clinical popularity of 35–45% peroxide systems and the ongoing debate around activation methods, an in vitro, standardized SEM–EDS comparison can clarify protocol-level differences and inform targeted remineralization strategies after bleaching [5,11,15,16]. Therefore, this experimental study compares three common chairside protocols under standardized conditions using SEM–EDS endpoints. The aim is to describe protocol-related surface and elemental shifts, not to rank products for clinical effectiveness.

## 2. Materials and Methods

### 2.1. Study Design

This in vitro study utilized human molars and premolars extracted for clinical purposes, adhering strictly to ethical standards. Approval for the research protocol was granted by the Bioethics Committee of the Victor Babeș University of Medicine and Pharmacy, Timișoara (Approval No. 09/11.03.2024). All procedures were carried out in accordance with the principles outlined in the Declaration of Helsinki and the guidelines for Good Practice in Biomedical Research.

To ensure meaningful statistical comparison between experimental and control groups, an a priori sample size calculation was performed using G*Power 3.1. The parameters included a significance threshold of α = 0.05, a statistical power of 0.80, and an anticipated effect size of 0.5, consistent with previous research on enamel alterations following bleaching procedures. Based on this calculation, a total of 42 specimens (n = 14 per group) was deemed necessary. To account for possible sample attrition or variability, an additional 20% was added to the initial sample pool.

Only molars and premolars were included. Incisors and canines were excluded due to low availability and increased curvature with thinner facial enamel, which complicates standardized, planar ROI acquisition at the magnifications used. Posterior teeth provided broader, flatter enamel surfaces, enabling reproducible sectioning, polishing, and ROI placement across matched halves.

Each tooth was sectioned longitudinally into two equal parts: one half was allocated as the control, while the other underwent bleaching treatment. The teeth were sourced from an anonymized biobank and processed in accordance with strict ethical protocols. Soft tissue remnants were carefully removed using periodontal curettes, and the samples were stored in distilled water for no more than one month to maintain hydration and preserve enamel characteristics. This storage method was performed to minimize dehydration-related bias and helps retain enamel properties that closely resemble in vivo conditions.

Specimens were examined under ×10 magnification (OMS2356, Zumax Medical Co, Ltd., Suzhou, Jiangsu, China), and any teeth presenting with fractures, carious lesions, previous restorations, or visible enamel defects were excluded from the study.

### 2.2. Preparation of Samples

A total of 42 human teeth were selected for inclusion in this study. Following the removal of any surface calculus, the teeth were immersed in a 0.1% thymol solution for five days to inhibit microbial growth. Subsequently, the crowns were separated 2 mm above the cementoenamel junction using a diamond disk under continuous water irrigation to prevent overheating. Each crown was then longitudinally divided along the cervical-occlusal axis, resulting in two symmetrical halves. These were labeled with the same identification code—one designated for experimental bleaching treatment and the other retained as a control counterpart, ensuring matched comparisons within each sample.

Each tooth half was embedded in self-curing acrylic resin (UNIFAST Trad, GC America) for stabilization and prepared for analysis through a standardized polishing protocol. Polishing was carried out using Soft-lex disks (3M ESPE, St. Paul, MN, USA) in a sequential manner: coarse (42 μm), medium (30 μm), and fine (15 μm) grits, each for a duration of two minutes. After each polishing stage, specimens were thoroughly rinsed with distilled water to eliminate residual particles.

Although artificial saliva is often employed to simulate oral conditions, in this study distilled water was used for storage due to limited access to standardized artificial saliva formulations. This choice was made to maintain sample hydration while avoiding uncontrolled remineralization effects, consistent with methodologies reported in similar in vitro studies.

### 2.3. Bleaching Protocol

The experimental specimens were subjected to in-office bleaching treatments using three commercially available whitening products frequently used in dental practice. Each bleaching agent was applied according to the manufacturer’s instructions. The products included one carbamide peroxide-based gel and two hydrogen peroxide-based systems, one of which required light activation.

- Carbamide Peroxide-Based Agent—Opalescence Quick (Ultradent Products Inc., South Jordan, UT, USA). This bleaching product contains 45% carbamide peroxide and is formulated as a ready-to-use gel, white in color, that does not require light activation. The gel was applied in a uniform 1–2 mm layer directly onto the enamel surface for a duration of 15 to 30 min per session. Its high peroxide concentration allows for short, in-office treatment protocols without prior mixing.
- Chemically Activated Hydrogen Peroxide Agent—Opalescence Boost (Ultradent Products Inc., South Jordan, UT, USA). This system contains 40% hydrogen peroxide and is delivered through a dual-syringe setup that ensures fresh mixing of the gel immediately before use. The red-colored bleaching gel is chemically activated and does not require light to initiate the whitening process. Application was performed in layers of 1–2 mm, with protocols consisting of one, two, or three consecutive 20 min applications (totaling up to 60 min per session), as recommended by the manufacturer.
- Light-Activated Hydrogen Peroxide System—BlancOne Ultra+ (IDS S.p.A., Savona, Italy). This product is based on 35% hydrogen peroxide and is supplied as a powder that must be manually combined with a liquid activator included in the kit. Once mixed, the resulting orange-colored gel is applied in 1–2 mm layers and activated with a dedicated light source for 8–10 min per application. Each bleaching session consisted of three light-activated cycles, immediately following gel preparation.

Before bleaching, all specimens were cleaned using a soft brush and distilled water to remove organic debris and ensure a clean enamel surface. After treatment, samples were stored in isotonic saline solution to prevent dehydration and preserve enamel structure. Representative enamel sections from each group were then prepared for Scanning Electron Microscopy (SEM) and Energy-Dispersive X-ray Spectroscopy (EDS) by sectioning and mounting on appropriate slides.

### 2.4. SEM and EDS Analyses

Surface morphology and elemental composition of enamel specimens were evaluated using Scanning Electron Microscopy (SEM) and Energy-Dispersive X-ray Spectroscopy (EDS). SEM analysis was carried out with an Inspect™ system operating in low vacuum mode (30 Pa), at an accelerating voltage of 15.0 kV and a working distance of 10.0 mm. Imaging was performed at a magnification of ×1100, with a field of view (FOV) of approximately 116.4 × 87.3 µm. A backscattered electron detector (BED-S) was employed to capture high-contrast surface details.

EDS analysis was conducted using the JSM-IT200 microscope (JEOL Ltd., Tokyo, Japan), also in low vacuum mode, under the same acceleration voltage of 15.0 kV. Spectra were collected from selected areas of each sample using a standardless ZAF (Ziegler–Armstrong–Fowler) quantification method. Each measurement had a live acquisition time of 30 s, a dead time of 2%, and a count rate of approximately 3500 CPS. Elemental analysis included quantification of major constituents such as carbon (C), oxygen (O), sodium (Na), magnesium (Mg), phosphorus (P), calcium (Ca), and traces of iron (Fe), where present.

Multiple regions of interest (ROIs) were analyzed per sample to ensure data consistency. The atomic and weight percentages obtained allowed assessment of surface compositional changes induced by the bleaching agents, complementing the morphological evaluation performed by SEM. These analyses aimed to identify demineralization patterns and variations in mineral content potentially associated with different bleaching protocols.

### 2.5. Statistical Analysis

Statistical analysis was performed using one-way ANOVA to compare the calcium and phosphorus content among the treated groups (Opalescence Quick, Opalescence Boost, BlancOne Ultra+). A significance level of *p* < 0.05 was applied. The results showed statistically significant differences in both calcium (*p* < 0.001) and phosphorus (*p* < 0.001) content between the treatment groups, indicating varying degrees of demineralization induced by each bleaching protocol.

## 3. Results

### 3.1. SEM Morphological Analysis

**Figure 1:**
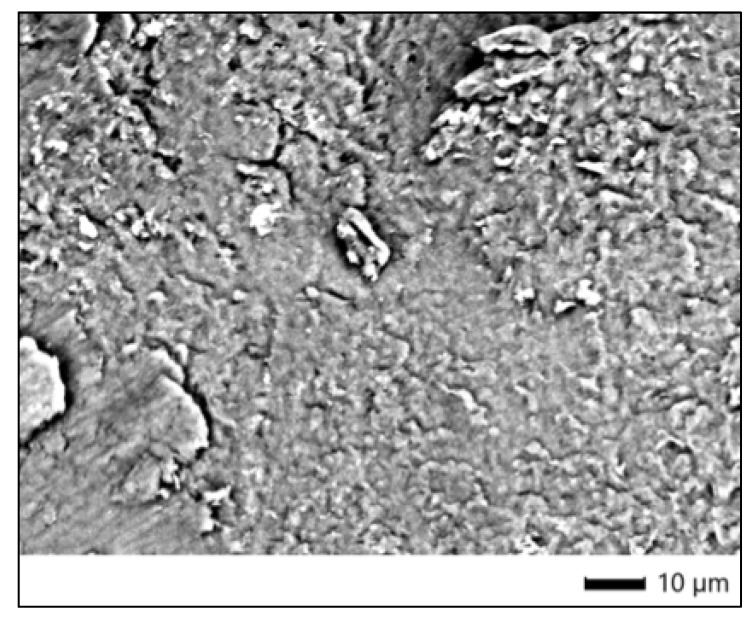
SEM micrograph of untreated enamel surface (control—Opalescence Quick).

SEM images of the control specimen from the Opalescence Quick group (3C), acquired at ×1100 magnification, revealed a relatively homogeneous enamel surface with moderate irregularities. The surface displayed a slightly granular texture, with areas of subtle surface roughness and minimal structural disruption. No pronounced prism dissolution, micro-cracking, or erosive defects were observed, suggesting that the untreated enamel retained its overall morphological integrity. These features are consistent with sound enamel morphology in in vitro conditions and serve as a reliable baseline for comparison with bleached samples (Figure 1).

### 3.2. Elemental Composition by EDS

EDS analysis of four distinct regions (Spc_009, Spc_010, Spc_011, and Spc_012) on the same specimen showed consistent elemental profiles representative of healthy enamel. The main elements identified and quantified were:

- Oxygen (O): Dominant in all spectra, with atomic percentages ranging from 44% to 50%, consistent with the presence of hydroxyapatite (Ca_10_(PO_4_)_6_(OH)_2_).
- Calcium (Ca): Ranged from 11.5% to 12.5% (atomic %), in line with the typical mineral content of enamel. These values suggest no significant demineralization in the control specimen.
- Phosphorus (P): Values varied between 7.5% and 8.9% (atomic %), supporting the presence of calcium phosphate compounds.
- Carbon (C): Detected in moderate amounts (26–33%), potentially associated with organic components, residual contaminants, or surface carbon coating.
- Sodium (Na) and Magnesium (Mg): Present in low but consistent concentrations (typically <2%), both elements are minor natural constituents of enamel.
- Iron (Fe): Observed only in one region (Spc_010) at 2.53% atomic, possibly due to external contamination or instrumental artifact.

The consistency across the four analysis points confirms the stability and uniformity of the enamel composition in the control sample. The preserved Ca/P ratio and absence of surface degradation in both SEM and EDS results confirm the expected characteristics of unbleached enamel, establishing a valid reference for evaluating subsequent bleaching-induced changes.

### 3.3. Morphological Assessment of Bleached Enamel

**Figure 2:**
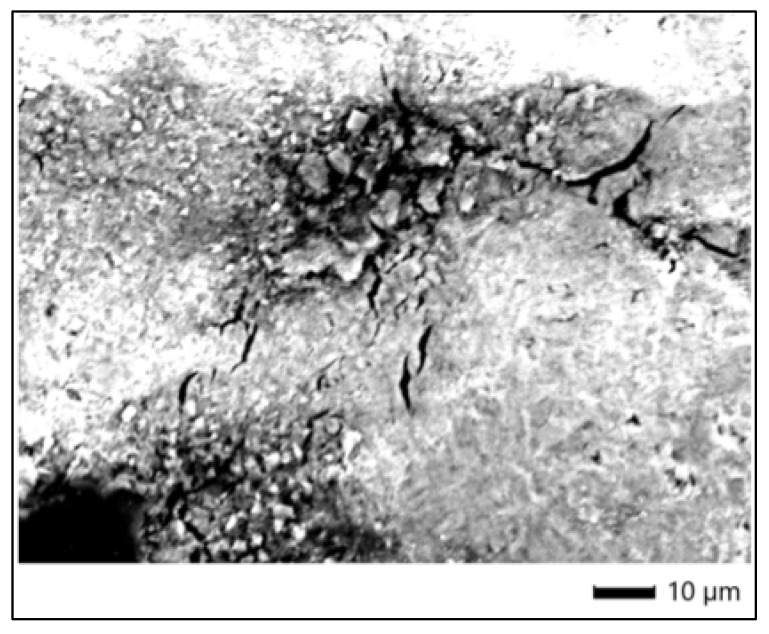
SEM micrograph of enamel treated with Opalescence Quick.

SEM imaging of the enamel surface following treatment with Opalescence Quick (sample 3P) revealed evident alterations in surface morphology when compared to the control (3C). The enamel exhibited a pronounced loss of surface uniformity, with distinct areas of roughness, fissures, and microcracks observable at ×1100 magnification. These morphological changes suggest surface degradation and structural stress potentially induced by the bleaching agent, even in the absence of light activation. The damage appeared localized in some regions, with crystal disintegration and surface fragmentation visible, particularly in areas of stress concentration (Figure 2).

### 3.4. Elemental Composition Changes After Bleaching

EDS spectra obtained from four regions (Spc_013, Spc_014, Spc_015, Spc_016) confirmed compositional variations relative to the control enamel:

- Oxygen (O): Maintained the highest atomic concentration (45–48%), consistent with the inorganic matrix.
- Calcium (Ca): Atomic percentages ranged from 9.53% to 11.73%, slightly lower than the control, indicating a possible mineral loss or surface demineralization.
- Phosphorus (P): Present in reduced concentrations (~7.41–8.59% atomic) compared to the control, suggesting hydroxyapatite breakdown.
- Carbon (C): Detected at 28–33%, showing no significant increase from control, suggesting limited organic contamination.
- Sodium (Na), Magnesium (Mg): Present in trace amounts, consistent with normal enamel composition.
- Chlorine (Cl): Detected in all regions (~0.18–0.20% atomic), likely introduced during the bleaching process or from preparation artifacts.
- Aluminum (Al) and Silicon (Si): Detected especially in Spc_014, with Al at 4.35% and Si at 11.92% atomic—these may indicate external contamination or remnants from polishing materials or the bleaching gel components.

The reduction in Ca and P atomic percentages, coupled with increased surface roughness and microcrack formation observed in SEM, supports the hypothesis that Opalescence Quick induces measurable chemical and morphological changes in enamel. While the absence of light activation may have moderated the impact compared to light-activated systems, the 45% carbamide peroxide concentration was sufficient to alter enamel integrity at a microstructural level.

### 3.5. SEM Morphological Evaluation

**Figure 3:**
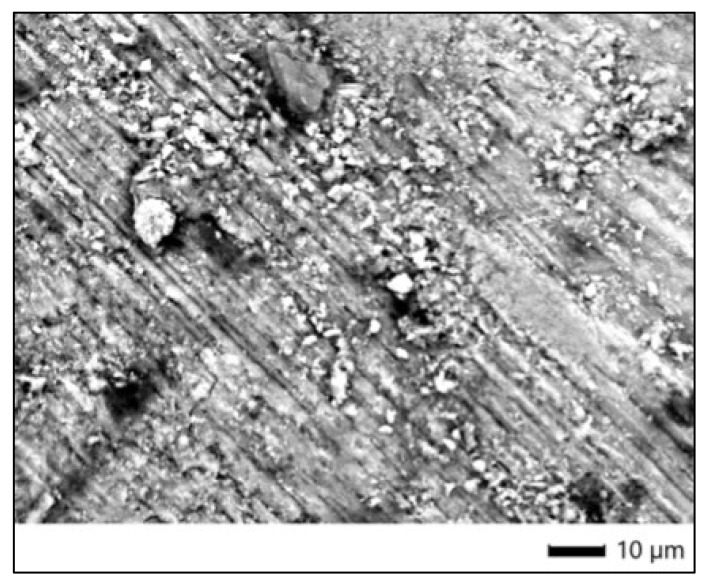
SEM image of control enamel surface (Opalescence Boost).

The SEM micrograph shows a relatively homogeneous enamel surface with evident polishing striations. There are dispersed particles likely originating from residual debris or environmental contamination, but no significant morphological alterations such as erosion, fissures, or etching are observed. The continuity and compactness of the enamel surface are characteristic of a non-treated control group. This serves as a stable baseline for comparison with bleached samples and confirms the sample’s structural integrity in the absence of oxidative or acidic exposure (Figure 3).

### 3.6. EDS Elemental Composition of Control Enamel

EDS analysis from the four measured points (Spc_001–Spc_004) indicates a stable and typical elemental composition for sound human enamel. The following elements were detected:

- Oxygen (O): Present in high amounts (31–45%), indicating the predominance of hydroxyapatite and confirming strong mineralization.
- Calcium (Ca): Ranges from approximately 13–28% by mass, consistent with normal enamel mineral content. This is essential for the structural integrity of enamel.
- Phosphorus (P): Detected at 7–15%, aligning well with stoichiometric expectations for hydroxyapatite (Ca_10_(PO_4_)_6_(OH)_2_).
- Carbon (C): Measured between ~15–25%, potentially due to the presence of organic components or superficial environmental carbon residues.
- Sodium (Na): Found in trace levels (~0.5–1.1%), which is typical for enamel and may influence its solubility.
- Magnesium (Mg): Minor quantities (~0.3–0.7%), known to substitute for calcium in enamel apatite and slightly affect its properties.
- Chlorine (Cl): Present at low levels (~0.2–0.3%), likely surface contamination.
- Silicon (Si) and Aluminum (Al): Detected only in one region (Spc_004), suggesting possible extrinsic contamination or sample holder residue.

The relatively low standard deviations and consistent Ca/P ratios across spectra confirm that the enamel chemistry is intact and unmodified, which reinforces the validity of this sample as a proper control reference in bleaching studies.

### 3.7. SEM Analysis—Bleached Samples

**Figure 4:**
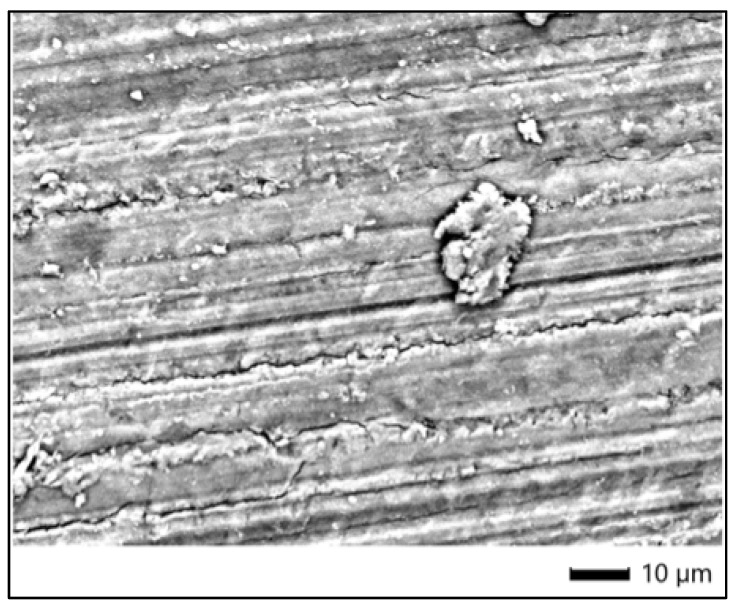
SEM image of enamel after bleaching with Opalescence Boost.

The enamel surface of the specimen treated with Opalescence Boost (sample 6P) showed notable alterations in surface morphology compared to the control. At ×1100 magnification, the SEM image revealed a relatively smooth surface with parallel polishing lines, but with the presence of dispersed surface deposits and granular accumulations. These deposits appeared clustered along abrasively modified areas and presented a more heterogeneous surface texture. Although no deep cracks or widespread erosive defects were visible, the enamel displayed signs of superficial restructuring, suggesting mild surface interaction with the 40% hydrogen peroxide bleaching agent used in this protocol (Figure 4).

### 3.8. EDS Analysis—Bleached Samples

Elemental composition assessed by EDS in four analyzed regions (Spc_001–Spc_004) indicated the following trends:

- Oxygen (O): Present at high atomic percentages (33.24–45.62%), oxygen remains the dominant element, reflecting the preserved inorganic matrix of the enamel.
- Calcium (Ca): Ranged from 13.80% to 27.94% (atomic), indicating variability in mineral content. Slight reductions in some areas may suggest early-stage demineralization.
- Phosphorus (P): Detected at 7.32–14.65% atomic, supporting the presence of calcium phosphate compounds, though some fluctuation between points was observed.
- Carbon (C): Present in moderate levels (15.51–25.17%), possibly reflecting organic residue or surface contamination; one of the values was noticeably lower than in the control.
- Sodium (Na): Found in trace amounts (0.50–1.06%), consistent with enamel’s natural composition.
- Magnesium (Mg): Ranged from 0.33% to 7.41% atomic, with a marked increase at one point (Spc_004), which may indicate local chemical imbalance or residual bleaching components.
- Aluminum (Al) and Silicon (Si): Detected in minor quantities only in Spc_002 and Spc_004, possibly from sample handling or polishing residue.
- Chlorine (Cl): Found in low concentrations (0.25–0.32%), potentially originating from bleaching by-products or residual preparation materials.

These findings indicate that Opalescence Boost, while not causing severe mineral loss, may induce localized compositional changes and surface modification, visible both morphologically and chemically. The overall enamel structure remains largely intact, although variations in Ca and P levels suggest some interaction with the bleaching agent.

### 3.9. BlancOne Ultra+ Group (SEM Morphological Evaluation)

**Figure 5:**
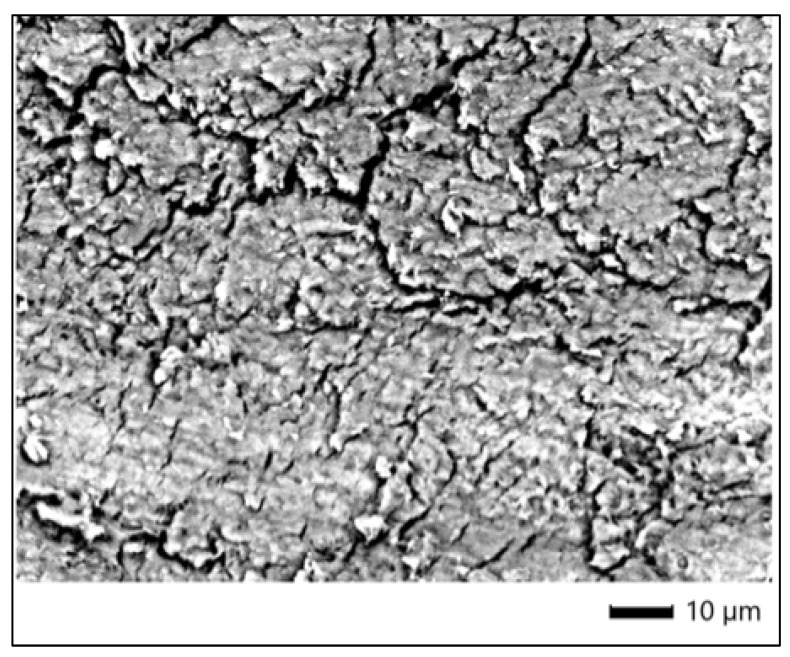
SEM micrograph of control enamel (BlancOne Ultra+).

SEM examination of the control specimen for the BlancOne Ultra+ group (9C) revealed a compact enamel surface with distinct morphological characteristics. At ×1100 magnification, the enamel exhibited a moderately rough texture, characterized by a dense network of superficial cracks and granular surface disruptions. These features suggest some degree of structural irregularity, possibly related to sample dehydration or pre-existing surface stress, but no aggressive material loss or prism disintegration was evident. Overall, the enamel surface preserved its structural continuity, indicating it had not been subjected to bleaching-induced degradation (Figure 5).

### 3.10. BlancOne Ultra+ Group (EDS Elemental Composition of Control Enamel)

EDS analysis was conducted in four different regions of interest (Spc_017 to Spc_020). The elemental profiles were consistent with sound, unbleached enamel and revealed the following key observations:

- Oxygen (O): The most abundant element, with atomic percentages ranging from 43.38% to 44.98%, reflective of the enamel’s hydroxyapatite structure.
- Calcium (Ca): Present in relatively high atomic concentrations (14.46% to 15.93%), indicating a well-mineralized surface.
- Phosphorus (P): Also consistent, with values around 9.27% to 10.22%, maintaining a stable Ca/P ratio across all measured points.
- Carbon (C): Ranged from 28.88% to 31.77% atomic, likely indicating the presence of residual organic content or environmental carbon contamination.
- Sodium (Na), Magnesium (Mg), and Aluminum (Al): Detected in low concentrations, each below 1% atomic, as expected for native enamel composition.

Fitting ratios: All values were below 0.05, confirming the reliability of the elemental quantification. The relatively high and uniform levels of Ca and P across all spots confirm a stable and intact mineral phase. Despite the presence of superficial cracks visible in SEM, the elemental distribution did not suggest significant mineral loss or chemical alteration. These findings validate the sample’s suitability as a control for comparison with bleached specimens.

### 3.11. BlancOne Ultra+ Group (SEM Surface Analysis-Bleached Samples)

**Figure 6:**
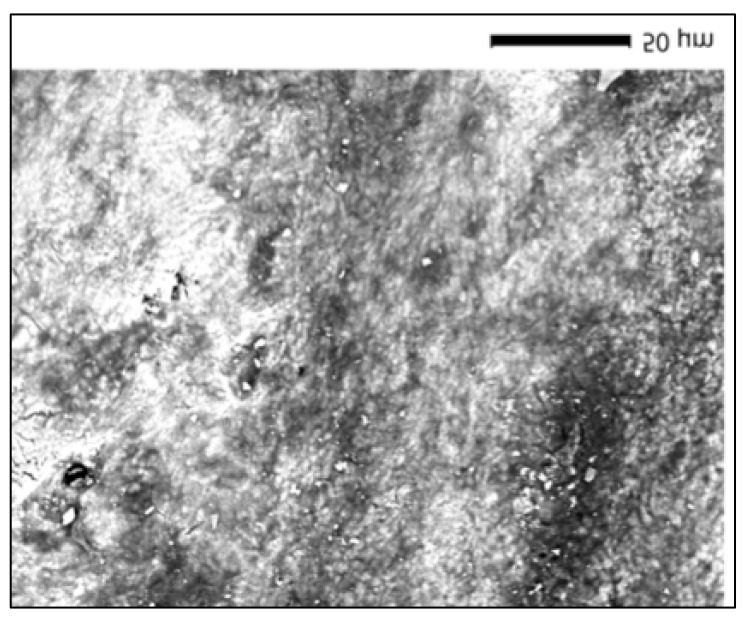
SEM micrograph of enamel after BlancOne Ultra+ bleaching.

SEM analysis of the enamel specimen treated with BlancOne Ultra+ (sample 9P) revealed noticeable surface alterations compared to its corresponding control (9C). At ×500 magnification, the enamel exhibited a heterogeneous morphology with areas of diffuse surface erosion and widespread irregularities. The surface appeared less defined, with smoothed features and loss of prismatic clarity. Numerous scattered deposits and darkened zones were visible, indicating structural modifications and possible mineral redistribution following the bleaching procedure. Unlike the control, surface continuity was disrupted, suggesting an alteration in surface mineralization likely associated with the high-concentration hydrogen peroxide and light activation employed in the BlancOne Ultra+ protocol (Figure 6).

### 3.12. BlancOne Ultra+ Group (EDS Elemental Composition Post-Treatment)

EDS analysis of five regions (Spc_021 to Spc_025) on the bleached enamel surface provided insights into compositional changes induced by the whitening process:

- Oxygen (O): Remained the most abundant element, with atomic percentages ranging from 39.20% to 45.42%, consistent with an oxygen-rich mineral matrix.
- Calcium (Ca): Showed a marked decrease in atomic concentration (as low as 1.42% in Spc_023 and 7.85% in Spc_025), indicating significant surface demineralization.
- Phosphorus (P): Also reduced, with values between 1.22% and 6.71% atomic, confirming partial loss of the hydroxyapatite component.
- Carbon (C): Relatively elevated in some areas, reaching up to 46.61% atomic in Spc_022, possibly indicating surface contamination or increased organic presence due to structural degradation.
- Sodium (Na) and Magnesium (Mg): Present in low amounts (<1% atomic), consistent with their natural trace levels in enamel.
- Aluminum (Al) and Silicon (Si): Detected particularly in Spc_023, possibly as residues from the bleaching agent or sample preparation.
- Potassium (K): Not typically present in natural enamel; detected only in Spc_023, suggesting potential contamination or external material deposition.

The notable drop in Ca and P levels, combined with morphological smoothing and disorganization seen in SEM, strongly suggests that the BlancOne Ultra+ bleaching agent had a pronounced etching and demineralizing effect on the enamel surface. These chemical and structural changes may reflect the impact of light-activated hydrogen peroxide bleaching at high concentration (35%) used in this group.

**Figure 7:**
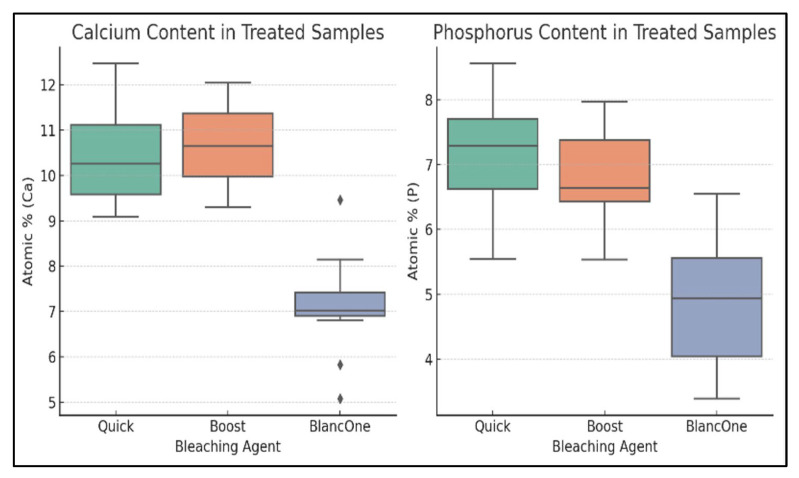
Comparative boxplot of elemental calcium and phosphorus (atomic %) in enamel samples following treatment with Opalescence Quick, Opalescence Boost, and BlancOne Ultra+.

Statistical comparison of EDS data from the treated enamel samples revealed significant differences in both calcium and phosphorus content across the three bleaching agents (*p* < 0.001 for both). BlancOne Ultra+ showed the lowest mean values for both elements, indicating the highest degree of surface demineralization, while Opalescence Quick preserved mineral content most effectively. These differences are visually summarized in the boxplots below, highlighting the variation in mineral loss among the whitening systems (Figure 7).

Across all protocols, oxygen remained dominant on EDS (~39–50 at%), while bleaching altered calcium and phosphorus to different extents consistent with SEM changes. In Opalescence Quick (45% CP), control enamel showed Ca 11.5–12.5 at% and P 7.5–8.9 at%, with homogeneous morphology; after bleaching, SEM revealed roughness, fissures, and microcracks, alongside modest mineral reductions (Ca 9.53–11.73 at%, P 7.41–8.59 at%) and trace Cl 0.18–0.20 at%; one spot contained Al 4.35 at% and Si 11.92 at%. Opalescence Boost (40% HP) controls were morphologically intact; EDS reported Ca 13–28 and P 7–15 by mass% (others within O 31–45%, C 15–25%), whereas bleached enamel showed mild surface restructuring and deposits with Ca 13.80–27.94 at% and P 7.32–14.65 at%; Mg rose locally to 7.41 at% (Spc_004), and Cl was 0.25–0.32 at%. BlancOne Ultra+ (35% HP, light-activated) controls had Ca 14.46–15.93 at% and P 9.27–10.22 at% despite superficial cracks; post-bleach, SEM documented diffuse erosion and loss of prismatic clarity, with pronounced demineralization (Ca 1.42–7.85 at%, P 1.22–6.71 at%) and C rising up to 46.61 at%; K appeared only in one spot (Spc_023). Overall, ANOVA across bleached groups showed significant Ca and P differences (both *p* < 0.001), with BlancOne Ultra+ exhibiting the lowest mineral values, Boost intermediate, and Quick the least change (Table 1).

Baseline EDS on the control halves showed no statistically significant differences in Ca or P between molars and premolars within each group (all *p* > 0.20; one-way ANOVA), supporting comparability of posterior tooth types for the present surface-level endpoints.

Within each 14-tooth group, molars predominated: Opalescence Quick—9/14 molars (64.3%) vs. 5/14 premolars (35.7%); Opalescence Boost—8/14 (57.1%) vs. 6/14 (42.9%); BlancOne Ultra+—10/14 (71.4%) vs. 4/14 (28.6%). Overall across all 42 teeth, molars comprised 27/42 (64.3%) and premolars 15/42 (35.7%), as seen in Table 2.

## 4. Discussion

This SEM–EDS experiment identified protocol-dependent, surface-level alterations under controlled, in vitro conditions. Morphologically, Quick-treated specimens exhibited roughness, fissures, and microcracks relative to their controls, while Boost produced mainly superficial restructuring with granular deposits and no deep cracking. In contrast, BlancOne Ultra+ yielded heterogeneous erosion, loss of prismatic definition, and disrupted surface continuity. These textural trends were mirrored chemically: in Quick, calcium fell from ~11.5–12.5 to ~9.53–11.73 atomic% and phosphorus from ~7.5–8.9 to ~7.41–8.59 atomic%; Boost showed localized variability (Ca 13.80–27.94 atomic%, P 7.32–14.65 atomic%) compatible with mild, region-dependent interactions; and BlancOne Ultra+ demonstrated marked demineralization with Ca as low as 1.42 atomic% and P as low as 1.22 atomic% in some ROIs, alongside elevated carbon signals (up to 46.61 atomic%). Group differences in Ca and P were significant (ANOVA *p* < 0.001), supporting a protocol-specific effect on surface mineral content.

Mechanistically, these findings align with differences in peroxide delivery and activation. Carbamide peroxide releases H_2_O_2_ more slowly (and at lower effective H_2_O_2_), likely moderating radical flux and limiting mineral loss despite visible microdefects. The chemically activated 40% H_2_O_2_ achieved whitening-relevant exposure yet produced only modest compositional shifts, consistent with controlled gel kinetics and neutral/slightly buffered formulations. The combination of high H_2_O_2_ and photoactivation in BlancOne Ultra+ plausibly increased transient radical availability and surface acidity/temperature at the gel–enamel interface, intensifying mineral dissolution within the EDS interaction depth. Trace detections of Cl (≈0.18–0.32 atomic%) and occasional Al/Si or K likely reflect residual reagents or preparation contaminants rather than true lattice constituents, but they underscore the sensitivity of the surface layer to exogenous inputs. Notably, the BlancOne control exhibited superficial cracking before treatment, which may have predisposed that group to greater post-bleach mineral loss at stress concentrators.

Clinically, the concurrence of rougher topography and reduced Ca/P proxies suggests a superficially demineralized, more reactive enamel immediately after certain chairside protocols—particularly light-activated systems. This supports pragmatic risk-mitigation: prefer neutral-pH, non-activated or chemically activated gels when esthetic goals allow; minimize total exposure time and number of cycles; interpose sessions; and pair bleaching with remineralization (fluoride varnish, CPP-ACP or calcium/phosphate gels) to hasten surface recovery. Because surface changes can influence bonding and stain uptake, delaying adhesive procedures or using antioxidants when immediate bonding is unavoidable may be prudent. Future work should couple SEM–EDS with microhardness/AFM roughness, pH/temperature logging during activation, and longitudinal remineralization trajectories in saliva-simulating media to define thresholds that preserve enamel while achieving target shade change.

The gradient of effects observed across protocols—least change with 45% CP, intermediate with chemically activated 40% HP, and the greatest surface/compositional disruption with light-activated 35% HP—tracks with clinical and laboratory evidence that photoactivation rarely improves whitening outcomes and may aggravate adverse effects. A meta-analysis of in-office bleaching found that adding light neither enhanced immediate or short-term color change with high-concentration HP nor reduced treatment time; conversely, it increased the odds of tooth sensitivity (OR ≈ 3.5) compared with non-light methods [17]. A later network meta-analysis reached a similar conclusion, ranking light-free, high-concentration HP among the most effective approaches without an added benefit from light, reinforcing that activation is not routinely warranted [18]. These data, together with the more pronounced roughness and Ca/P depletion under light activation, support favoring chemically activated or non-activated chairside gels when possible.

Protocol chemistry helps explain the differential SEM–EDS patterns. In vitro, altering gel pH does not reliably change color efficacy, but enamel microhardness falls immediately after a single session regardless of whether 35% HP is acidic, neutral, or alkaline, indicating that radical flux and exposure time dominate over pH within common ranges [19]. Earlier work similarly showed that more acidic HPs increase roughness and affect surface properties more than neutral formulations, even when color outcomes remain comparable [20]. The modest compositional shifts with 40% HP and the stronger demineralization with light-activated 35% HP are consistent with protocols that either limit radical availability (neutral/chemically activated) or transiently amplify it at the gel–enamel interface (photoactivation), respectively, producing the morphology–composition coupling you measured.

The EDS drops in Ca and P—extreme in some ROIs after light activation—align with studies that have quantified mineral loss and Ca/P ratio changes after peroxide exposure. Using EDS, Llena et al. showed that both HP and CP can reduce Ca and P and alter prismatic definition, while control enamel maintains a stable Ca/P profile [21]. At the same time, surface-protective strategies can blunt these losses: pre-bleach brushing with bioactive glass or arginine-carbonate toothpastes preserved Ca/P and yielded smoother post-bleach surfaces versus controls in an Operative Dentistry study (EDS + SEM endpoints) [22]. Beyond pre-treatment, modifying gels intrinsically with remineralizers can also help; adding Ca and/or F to HP significantly reduced enamel microhardness loss and peroxide diffusion without compromising ΔE* color change in vitro [23]. These reports substantiate these finding that systems differ in demineralization potential and suggest specific levers—pretreatment and gel additives—to mitigate surface mineral depletion.

Post-bleaching recovery pathways further contextualize these results. After a high-concentration HP session, microhardness commonly falls by ~15–20% and SEM shows more irregularities; in a controlled study (35% HP), mean Vickers hardness dropped ≈18% but recovered toward baseline (↑16–33%) after applying remineralizing agents, with visible superficial mineral deposition on SEM [24]. Newer factorial experiments indicate that topical NaF applied before/during/after bleaching restores microhardness at multiple subsurface depths (20–120 µm) without diminishing whitening effectiveness, outperforming some calcium-phosphate formulations in this respect [25]. In practice, these data endorse pairing chairside sessions with immediate fluoride (e.g., neutral 2% NaF) and/or short courses of CPP-ACP/F-containing products to accelerate re-hardening of the reactive, Ca/P-depleted surface EDS detected.

Finally, this observation of roughness and microcracks has procedural implications for adhesive dentistry. Residual oxygen from bleaching can transiently inhibit free-radical polymerization and depress resin-enamel bond strength; the classic reversal with 10% sodium ascorbate confirms a redox-driven mechanism, and delaying bonding by ~1–2 weeks is often sufficient to normalize adhesion in vitro [26]. Coupled with evidence above that activation does not improve efficacy, a conservative clinical bundle emerges from the observed data and the literature: prefer neutral-pH, non- or chemically activated gels; minimize total exposure time/cycles; apply fluoride or CPP-ACP immediately post-bleach; and schedule adhesive procedures after a short waiting period or use antioxidants when immediacy is unavoidable [17,18,19,22,23,24,25,26].

Interpretation should remain conservative: these findings are descriptive of early surface changes under the specific protocols tested and do not constitute comparative clinical efficacy.

Regarding study limitations, this is an in vitro, surface-focused assessment; hence the absence of acquired pellicle, dynamic salivary buffering, and masticatory thermomechanics limits generalization. Distilled-water storage (rather than standardized artificial saliva) may over- or underestimate post-treatment remineralization. EDS offers semi-quantitative data within a shallow interaction volume and used standardless ZAF; localized heterogeneity (e.g., a Mg spike to 7.41 atomic% in one ROI) and occasional contaminants (Al/Si/K/Cl, high carbon) highlight potential preparation or measurement artifacts. Baseline microcracking in the BlancOne control could have amplified subsequent damage, and ROI selection at ×500–×1100 magnification may bias local chemistry. Because only posterior teeth were included, generalization to anterior enamel (thinner, more curved, with differing prism exposure) should be cautious; nonetheless, control-side Ca and P did not differ between molars and premolars under our ROI protocol. Finally, differences in manufacturer-directed cycle counts and unmeasured gel pH/irradiance confound direct attribution of effects solely to peroxide concentration or activation.

## 5. Conclusions

Within the constraints of an in vitro, surface-focused assessment, all three protocols yielded morphological and compositional alterations detectable by SEM–EDS, with a severity gradient (BlancOne Ultra+ > Boost > Quick). These data should not be interpreted as clinical hierarchies; rather, they underscore the need for neutral-pH, non- or chemically activated regimens and prompt remineralization when feasible, while recognizing that in-mouth conditions may mitigate or amplify surface effects.

## Figures and Tables

**Table 1:** SEM–EDS summary of enamel surface and elemental composition before/after each bleaching protocol.

Group/Protocol	Specimen	SEM Morphology (Magnification)	O (%)	Ca (%)	P (%)	C (%)	Other Elements (Notes)	EDS Units
Opalescence Quick (45% CP; no light)	Control	Homogeneous enamel; slight granular texture; minimal roughness; no cracks/erosion (×1100).	44–50	11.5–12.5	7.5–8.9	26–33	Na, Mg <2%; Fe 2.53 (Spc_010).	Atomic %
Opalescence Quick (45% CP; no light)	Bleached	Loss of surface uniformity; roughness, fissures, microcracks; localized crystal disintegration (×1100).	45–48	9.53–11.73	7.41–8.59	28–33	Na, Mg traces; Cl 0.18–0.20; Al 4.35 and Si 11.92 (Spc_014).	Atomic %
Opalescence Boost (40% HP; chemically activated)	Control	Homogeneous surface with polishing lines; no erosion/fissures; stable baseline (×1100).	31–45	13–28	7–15	15–25	Na 0.5–1.1; Mg 0.3–0.7; Cl 0.2–0.3; Si and Al only in Spc_004.	Ca, P by mass %
Opalescence Boost (40% HP; chemically activated)	Bleached	Smooth with parallel lines; dispersed surface deposits/granular accumulations; mild superficial restructuring (×1100).	33.24–45.62	13.80–27.94	7.32–14.65	15.51–25.17	Na 0.50–1.06; Mg 0.33–7.41 (spike at Spc_004); Al and Si minor (Spc_002, Spc_004); Cl 0.25–0.32.	Atomic %
BlancOne Ultra+ (35% HP; light-activated)	Control	Compact surface; dense superficial cracks/granular disruptions; no aggressive material loss (×1100).	43.38–44.98	14.46–15.93	9.27–10.22	28.88–31.77	Na, Mg, Al <1%.	Atomic %
BlancOne Ultra+ (35% HP; light-activated)	Bleached	Heterogeneous surface with diffuse erosion; loss of prismatic clarity; scattered deposits; disrupted continuity (×500).	39.20–45.42	1.42–7.85	1.22–6.71	up to 46.61	Na, Mg <1%; Al and Si present (esp. Spc_023); K detected only in Spc_023.	Atomic %

CP—carbamide peroxide; HP—hydrogen peroxide; SEM—scanning electron microscopy; EDS—energy-dispersive X-ray spectroscopy.

**Table 2:** Tooth-type distribution by group (n per group = 14).

Group	Molars (n)	Premolars (n)
Opalescence Quick	9	5
Opalescence Boost	8	6
BlancOne Ultra+	10	4

## Data Availability

Data availability are subject to hospital approval.
